# ^13^C CPMAS NMR as an Alternative Method to Verify the Quality of Dietary Supplements Containing Curcumin

**DOI:** 10.3390/molecules28083442

**Published:** 2023-04-13

**Authors:** Paweł Siudem, Łukasz Szeleszczuk, Agnieszka Zielińska, Katarzyna Paradowska

**Affiliations:** Department of Organic and Physical Chemistry, Faculty of Pharmacy, Medical University of Warsaw, Banacha 1, 02-097 Warsaw, Poland

**Keywords:** curcumin, dietary supplements, NMR, GIPAW, PXRD

## Abstract

Turmeric is a traditional Indian spice that has recently become very popular worldwide because it contains a powerful ingredient called curcumin, which has strong anti-inflammatory properties. Hence, dietary supplements containing extracts rich in curcumin have gained great popularity. The main problems related to curcumin-containing dietary supplements are poor water solubility and the fact that they are often faked by using synthetic curcumin instead of the plant extract. In this article, we propose the use of the ^13^C CPMAS NMR method to control the quality of dietary supplements. The analysis of ^13^C CPMAS NMR spectra supported by GIPAW computations allowed us to identify a polymorphic form present in dietary supplements (which affected the solubility of curcumin) and to point out a dietary supplement that could be faked by using synthetic curcumin. Further PXRD and HPLC investigations confirmed that the examined supplement contained synthetic curcumin instead of the genuine extract. Our method can be used for routine control, especially because the investigation is performed directly from the capsule/tablet content and does not require any special sample preparation.

## 1. Introduction

Until recently, curcumin (CUR) was treated merely as an orange pigment responsible for the intensive yellow color of the spice known as turmeric, being one of many spices. Today, on the basis of numerous investigations performed worldwide, we know that it is one of the strongest substances with an anti-inflammatory activity [1,2,3,4,5,6]. By its anti-inflammatory and antioxidation activity, it also exhibits antirheumatic properties [7,8,9]. Moreover, it was also found to exhibit an anticancer activity [10,11,12,13,14,15,16,17,18]. The Food and Drug Administration (FDA) and European Food Safety Authority (EFSA) have recognized curcumin as a substance safe for humans, based on the data from performed clinical studies and available publications [19,20,21]. Curcumin is a substance of enormous therapeutic potential, exhibiting no serious side effects but with very low water solubility [22]. In the future, it could become an alternative to the drugs currently available on the market. All this explains the huge popularity of curcumin as a component of dietary supplements. 

A dietary supplement is defined as any vitamin/mineral substance or an added chemical material, herbal product, botanical, amino acid, or any other foodstuff that is introduced into the diet to improve human health. Supplements are universally used and represent a wide range of products [23]. The market of these products is presently rapidly developing. Their great popularity is mostly due to their general availability (you can buy them in drugstores, herbal stores, or online shops), their global presence in mass media, and a relatively easy procedure to introduce them into the market. Concerning the safety of dietary supplements, it should be remembered that first of all, they should contain no impurities and forbidden substances; their quality and the real value of the declared components are also important, as in the case of curcumin and its extract. The selective control of the market and the lack of detailed guidelines concerning the parameters of different forms of supplements leave considerable freedom to the manufacturers, at the same time permitting them to launch on the market products of dubious quality that can even be dangerous to consumers by being completely devoid of a pro-health activity. In view of the growing interest of consumers in this group of products, in this work we present a method of fast evaluation of the quality of the extract present in dietary supplements.

As yet, curcumin has not been registered as a medicinal substance. However, there are many curcumin-containing dietary supplements available on the market. A study of the quality of such supplements is a very important analytical task of practical aspects [24]. In order to analyze the dietary supplements containing curcumin, it is worth applying the results obtained from structural investigations using nuclear magnetic resonance. ^13^C CPMAS solid-state NMR (ssNMR) is commonly used in pharmaceutical analyses [25,26]. Previously, a few studies reported its application in distinguishing between different polymorphic forms of API present in final pharmaceutical dosage forms [27,28,29]. It allows the analysis of the polymorphic form in the final product due to the possibility of the conversion from one form to another. It is highly important in the case of slightly soluble substances or when one polymorphic form has fundamentally better or lower solubility than others (see the case of ritonavir [30,31]). Curcumin has three described polymorphic forms differing in solubility in water, with Form I being the worst soluble [22]. DFT calculations support NMR analyses and are frequently used in combination with NMR spectra interpretations [32,33,34,35]. The GIPAW method includes the periodic boundary conditions of a studied crystal lattice, unlike the still-popular GIAO method [36].

Thanks to earlier studies (three polymorphic forms deposited in The Cambridge Structural Database (CSD) [37]), it was possible to determine which of the polymorphic forms of curcumin was present in the analyzed supplements. The aim of the study was to show that NMR spectroscopy could be used to carry out a standard analysis of the composition and quality of foodstuff, the purpose of which was to supplement a normal diet, known as dietary supplements. Curcumin-containing preparations were used in this study as an example.

## 2. Results and Discussion

**Figure 1:**
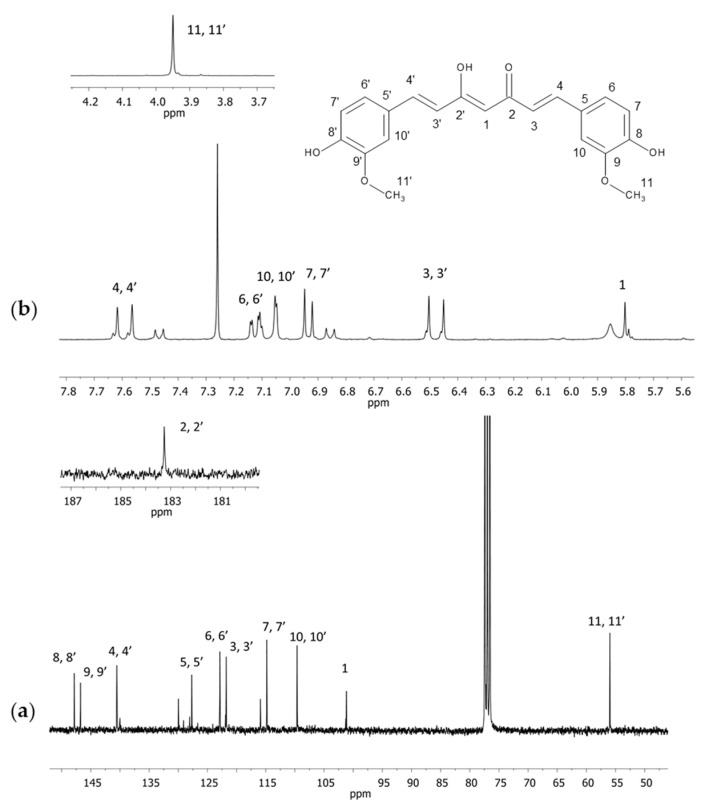
NMR spectra of curcumin in CDCl_3_ (**a**) ^13^C and (**b**) ^1^H with an extended section of 4.7–4.2 ppm (**a**) and 181–187 ppm (**b**). Numbers correspond with carbon or hydrogen atoms in curcumin structure.

The ^1^H and ^13^C NMR spectra in the CDCl_3_ and ^13^C CPMAS spectra of curcumin were acquired. In the spectra recorded in CDCl_3_ (both in ^1^H and ^13^C NMR), other signals were visible apart from the signals of curcumin (Figure 1). Their occurrence was probably related to the purity of the examined substance. 

The applied standard contained over 65% pure curcumin, but also its derivatives demethoxycurcumin and bisdemethoxycurcumin were present. Hence, for purification purposes, a recrystallization from isopropanol was performed. The choice of the solvent was due to the fact that the two main derivatives of curcumin were more easily dissolved when cold in isopropanol than the curcumin itself (Table 1) [38].

**Figure 2:**
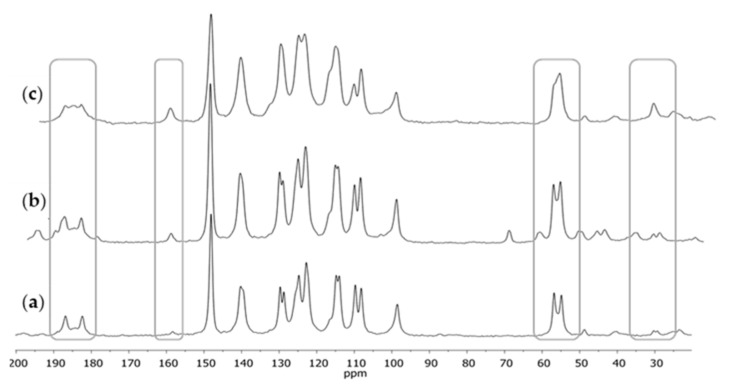
The ^13^C CPMAS NMR spectra of curcumin: (**a**) after crystallization from isopropanol, (**b**) before crystallization, and (**c**) the residue after crystallization. The differences at the key chemical shifts are marked on spectra.

After crystallization, a series of ^13^C CPMAS NMR spectra was registered again; of the substance before purification, then after purification, and of the residue after solvent evaporation to crystallization (Figure 2). After crystallization, narrower signals occurred in the spectra, with a better-shaped structure of double signals. In the case of residue after crystallization (Figure 2c), which mainly contained demethoxycurcumin and bisdemethoxycurcumin, the signals were much broader; in the case of a few signals (e.g., those from carbonyl carbons C2 or C2’ or methoxyl carbons C11 or C11’), no clear double signal was visible, which could be observed in the case of the ^13^C CPMAS NMR spectra for CUR.

**Figure 3:**
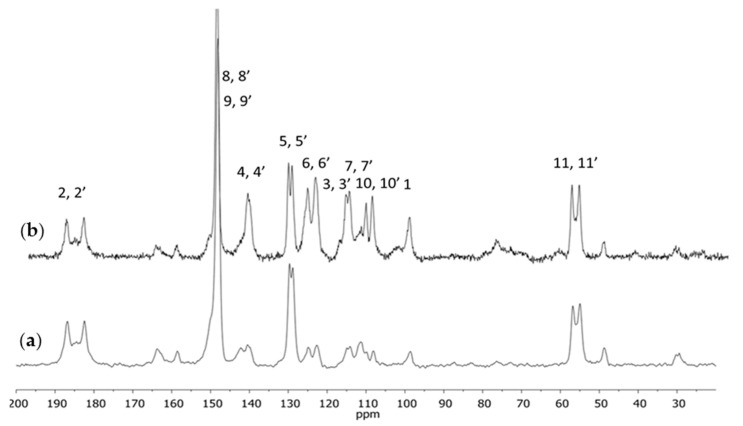
The ^13^C CPMAS NMR spectra of curcumin with the dipole filter (**a**) and standard (**b**).

The purified curcumin after crystallization was used for further investigations. The spectra presented in Figure 3 corresponded with the standard ^13^C CPMAS NMR experiment and with the experiment using a dipole filter. A reduced intensity of the signals originating from the CH and CH_2_ groups could be seen whereas the intensity of the signals originating from the quaternary carbon atoms and the methoxyl group remained unchanged. 

The greatest differences in chemical shift values between the spectra in the solution and in the solid state were observed for the carbon atoms C1 and C2 (C2’) as well as C5 (C5’) and C6 (C6’). They corresponded, respectively, with the carbon atom with the attached acidic proton (C1), the C=O groups (C2 and C2’), and also the quaternary carbon atom from the aromatic ring attached to the aliphatic fragment (C5 and C5’) and the vicinal aromatic carbon (C6 and C6’). The doubling of practically all signals indicated that, in the examined sample, two different and not-overlapping conformations of curcumin were present side by side. In the case of double signals C2 (C2’) and C5 (C5’), considerable differences between the chemical shift in the solution and solid state could be observed for only one of the components of the ^13^C CPMAS NMR divided signal. The greatest differences for the signals C1 and C2 (C2’) may have been related to the engagement of this molecule fragment in the formation of intermolecular hydrogen bonds whereas the differences in the aromatic ring may have resulted from the arrangement of molecules with respect to each other, or perhaps from the “twisting” of one of the rings with regard to the chain, which did not occur in the solution. In order to better know the reasons, an analysis using theoretical calculations for curcumin molecules was performed, described in a later part of the paper.

In the literature, three polymorphic forms of curcumin have been described so far [37,39]. In order to establish which of those forms was present in the examined sample, GIPAW computations were performed for periodic systems, taking into account the three forms deposited in The Cambridge Structural Database [37]. The structures present in the database are shown in Table S1 in Supplementary Materials.

Based on the analysis of parameters of the individual results from The Cambridge Structural Database, they were classified into three polymorphic forms. Most of them, as many as eleven results (BINMEQ, BINMEQ1, BINMEQ2, BINMEQ3, BINMEQ4, BINMEQ5, BINMEQ9, BINMEQ10, BINMEQ11, BINMEQ13, and BINMEQ14), corresponded with Polymorph I. The measurements were performed at different temperatures, but the unit cell parameters were comparable. BINMEQ6, BINMEQ8, and BINMEQ12 corresponded with Polymorph II whereas BINMEQ7 corresponded with Polymorph III. 

For each polymorphic form, one result was selected for the calculations. As BINMEQ5, BINMEQ6, and BINMEQ7 were registered at the same temperature, they were chosen to be the representatives of the particular polymorphs. Thus, it was possible to reduce the effect of temperature on the obtained unit cell parameters.

The first step in the computations was to choose the appropriate functional, which could affect the obtained results. Therefore, computations were performed using such functionals as the general gradient approximation (GGA); the PBE (Pedrew–Burke–Ernzerhof) exchange-correlation functional, taking into account Grimme’s or Tkatchenko–Scheffler (TS) dispersion corrections; the Pedrew–Wang potential (PW91) with the Ortmann–Bechstedt–Schmidt (OBS) correction; and the Wu–Cohen (WC), the modified PBE (RPBE), or PBESOL for the solid state.

According to our knowledge, GIPAW computations have not been published for all three polymorphic forms. They were performed for Polymorphs I and II in order to verify the assignment of chemical shift values in ssNMR [39]. Matlinska et al. carried out GIPAW calculations for I and II polymorphic forms [40], employing OTFG pseudopotentials. The obtained chemical shift values presented for Polymorph II were comparable with our results. The calculated chemical shifts for Polymorph I are not included in the manuscript or in the Supplementary Materials. The results of several investigations were published in which the DFT calculations were performed for an isolated molecule in a vacuum [41]. According to our knowledge, no computations for Polymorph III have been performed so far, and computation results have not been applied to the analysis of dietary supplements.

The computations using ten different methods were performed for BINMEQ5. The best functional was chosen based on the results of Polymorph I only, because the calculations for Polymorphs II and III required much more time and computing power. The obtained unit cell parameters for BINMEQ5 using different functionals are presented in Table 2.

A criterion of choosing the best functional was to find the best possible match of the value of the unit cell volume with data from the crystallographic database. In Table S1, which presents all the results present in the database whilst searching for the curcumin molecule, it can be observed that with the decrease in measurement temperature, the unit cell volume was slightly reduced. That is why, as the volume of BINMEQ7 was equal to 1759.76 Å^3^, the result obtained in the calculations (for a temperature of 0 K) should have been a little below that value.

The greatest differences between the theoretical and experimental values occurred in the case of functionals GGA RPBE (2973.8 Å^3^), GGA WC (2411.54 Å^3^), and GGA PW91 (2391.67 Å^3^). The closest result was obtained for GGA PBE TS (1755.64 Å^3^), which, according to expectations, was a little smaller than the experimental value (1759.76 Å^3^). Hence, this functional was chosen as the best one for periodic calculations; the system optimization and the calculation of the NMR parameters. The obtained theoretical results in comparison with the experimental ones are presented in Table 3.

In order to better illustrate the calculated chemical shift values for particular polymorphs and those obtained experimentally, Table 4 shows the differences between the theoretical and experimental results. Neither of the simulated spectra fully mapped the spectrum of the examined substance. However, the spectrum corresponding with Polymorph I was the most similar.

When we compared the chemical shift values collected in Table 4, it could be seen that the smallest differences between the experimental and theoretical values occurred for Polymorph I. This was particularly visible in the case of carbon atoms C1, C6, and C6` where the differences for Polymorphs II and III were much bigger than those for Polymorph I (Table 4). The key signal that made it possible to distinguish Polymorph I from the other ones was the signal originating from carbon atom C1 (the central atom in the molecule). In the case of Polymorph I, it occurred in the spectrum at the chemical shift value of below 100 ppm (δ of about 98 ppm) whereas for the other two polymorphs, it was in the range of δ 105–107 ppm. Thus, this signal could be acknowledged as the key diagnostic signal to distinguish Polymorph I from the remaining ones.

**Figure 4:**
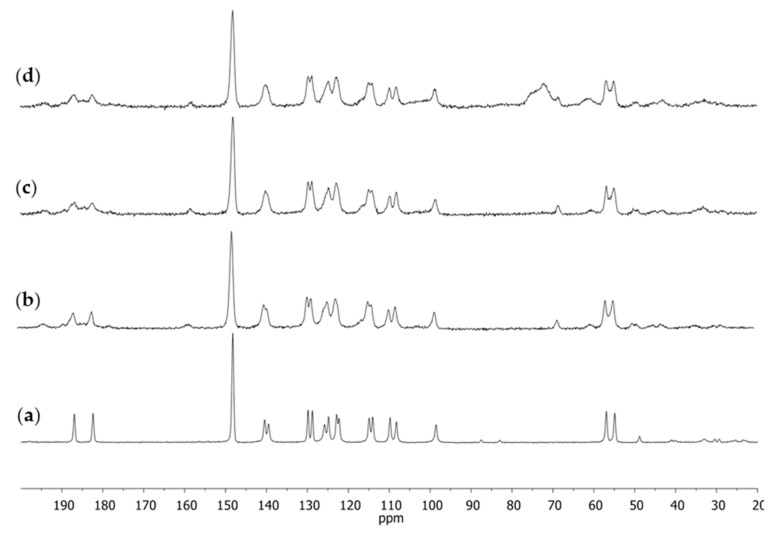
The ^13^C CPMAS NMR spectra of dietary supplements: (**a**) S1; (**b**) S2; (**c**) S3; (**d**) S4.

The obtained results for the substance assumed to be the standard induced us to use the same approach when analyzing complex products such as dietary supplements. That is why the next stage of our studies was to apply NMR spectroscopy to investigations of curcumin-containing dietary supplements. The ^13^C CPMAS NMR spectra of four selected dietary supplements were registered. Three of them were in the form of capsules (S1–S3) and one (S4) was in the form of a tablet. The obtained spectra are shown in Figure 4.

On the basis of earlier conclusions, it could be supposed that in all dietary supplements, curcumin occurred as Polymorph I because in each of the ^13^C CPMAS NMR spectra there occurred a single signal from the C1 carbon atom at δ of about 98 ppm.

**Figure 5:**
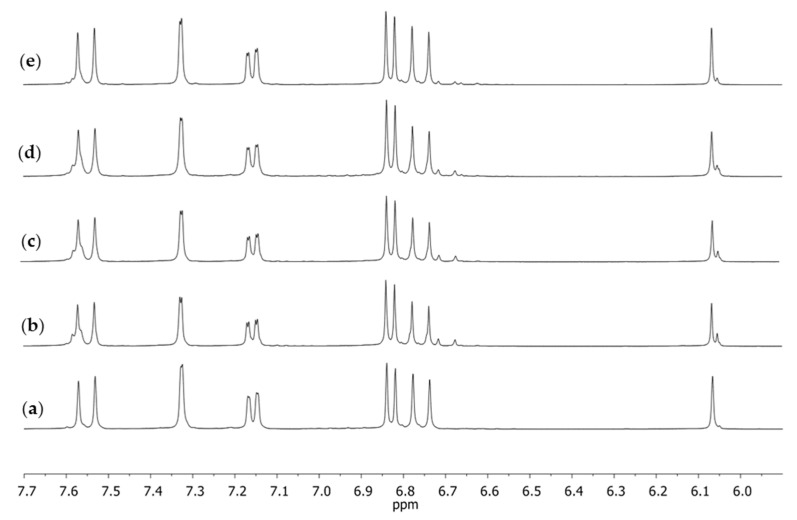
1H NMR spectra in DMSO-*d*_6_ of dietary supplements, the aromatic fragment obtained for (**a**) S1, (**b**) S2, (**c**) S3, (**d**) S4, and (**e**) curcumin; the reference substance after crystallization from isopropanol.

The ^13^C CPMAS NMR spectrum for supplement S1 (Figure 5a) considerably differed from the remaining ones. All the signals were narrower than in the other spectra and, concerning the doublets, they were better resolved than in the other dietary supplements examined. This may have been due to the fact that this sample was more crystalline [42]. Concerning the other products, their signals were broader and comparable with the spectrum of the pure substance. In the case of the only tablets examined (Figure 5d), in the spectrum there was also a very broad signal at δ of about 70 ppm. In this range, the filling substances of the tablet such as cellulose or lactose could be seen, or the polymers forming the shell [43]. As the signal was very broad and so single components could not be observed, the auxiliary substances present in the tablet could not be identified either.

If we compared the ^13^C CPMAS NMR spectra of the four examined dietary supplements in relation to pure curcumin after recrystallization, it could be observed that the spectrum visible in Figure 5a was considerably different from the other ones as well as from the pure substance. The extremely narrow and well-resolved signals indicated that the examined supplement had a more crystalline form than the remaining ones. However, it was interesting to observe that the recrystallized standard substance purified from curcumin derivatives provided a ^13^C CPMAS NMR spectrum with broader signals than those of the supplement for which the spectrum was performed straight from the capsule content. It seemed evident that the plant extract should be less purified and more amorphic than the pure standard substance.

In view of the obtained results and the information that in curcumin-containing dietary supplements, the manufacturers happened to substitute artificial curcumin (because of its lower price) for the genuine one, it is worth considering the assumption that the examined supplement might contain such artificial curcumin. The chemical synthesis performed in the lab aimed to selectively obtain one product with a high yield whereas in the plant, there are numerous secondary metabolites and, apart from the main product, at least several of its derivatives were present.

By the ^13^C CPMAS NMR method, it was impossible to unequivocally state if the obtained spectrum showed only the crystallinity of the sample or if it also pointed to its purity, quality, or technology of fabrication. However, when we dissolved the sample and performed the experiment in a solution, the sample crystallinity no longer had any effect on the spectrum appearance whereas the number of resonance lines and their shape could have been related to the purity of the sample. That is why, for each of the supplements, the ^1^H NMR spectra were registered after dissolving them in DMSO-*d*_6_. The same spectrum was also registered for the curcumin standard after crystallization (Figure 5).

**Figure 6:**
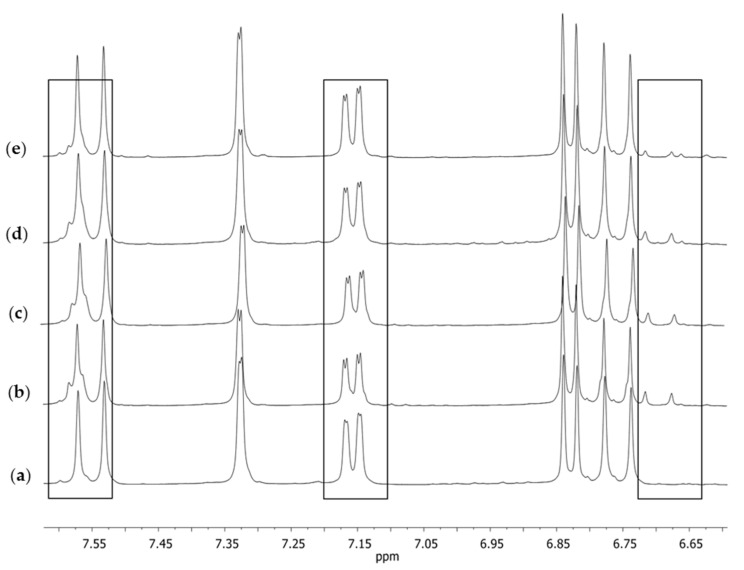
The extended aromatic fragment of the ^1^H NMR spectra in DMSO-*d*_6_ obtained for (**a**) S1, (**b**) S2, (**c**) S3, (**d**) S4, and (**e**) curcumin; the standard substance after crystallization from isopropanol.

Similarly, as in the case of the spectrum in the solid phase, the spectrum obtained in the solution for S1 (Figure 5a) was different from the other spectra. Both in the case of the reference substance (Figure 5e) and the remaining supplements (Figure 5b–d), at a signal (singlet) of the chemical shift of δ 6.07 ppm, which corresponded with the proton at C1, there was a small signal on its right-hand side. No such signal was present in the case of supplement S1. Several other signals of small intensity could also be observed, which may have pointed to the presence of other compounds accompanying natural curcumin that did not occur in the spectrum of supplement S1. These signals were visible in the extended fragment of the aromatic range (Figure 6). They were characterized by a low intensity in the range of 6.65–6.75 ppm, a signal on the slope of the signal at δ 7.60 ppm, and a clear doubling of the doublet components (7.15 ppm), which were only lightly outlined in the case of supplement S1 (Figure 6a). 

**Figure 7:**
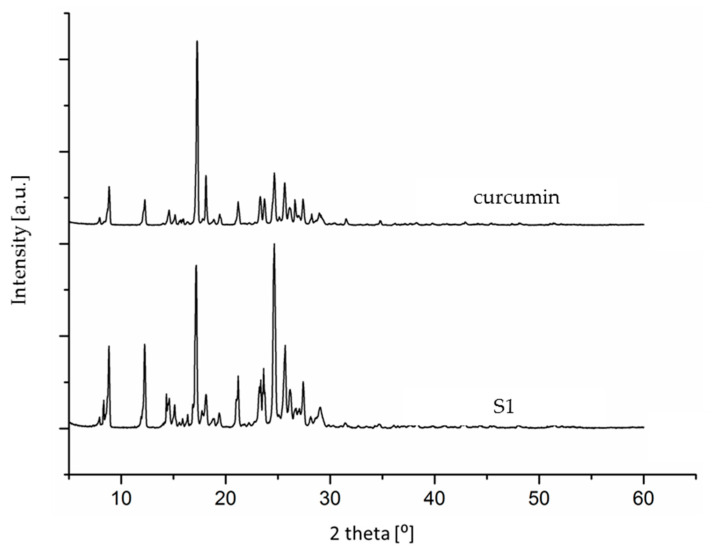
The PXRD diffractograms for the S1 supplement and the standard substance (after crystallization from isopropanol).

The analysis of the ^1^H NMR spectra of the examined supplements suggested that our earlier supposition that one of the supplements might contain pure synthetic curcumin (i.e., be faked) could be true. This was additionally confirmed by the obtained diffractograms (using powder X-ray diffraction (PXRD)). In order to compare and observe certain differences, the crystallization of curcumin was also performed directly from the dietary supplement. The obtained diffractograms for the crystallized curcumin standard and the crystallized supplement (S1) differed from each other, which also confirmed a different origin of the two samples (Figure 7).

**Figure 8:**
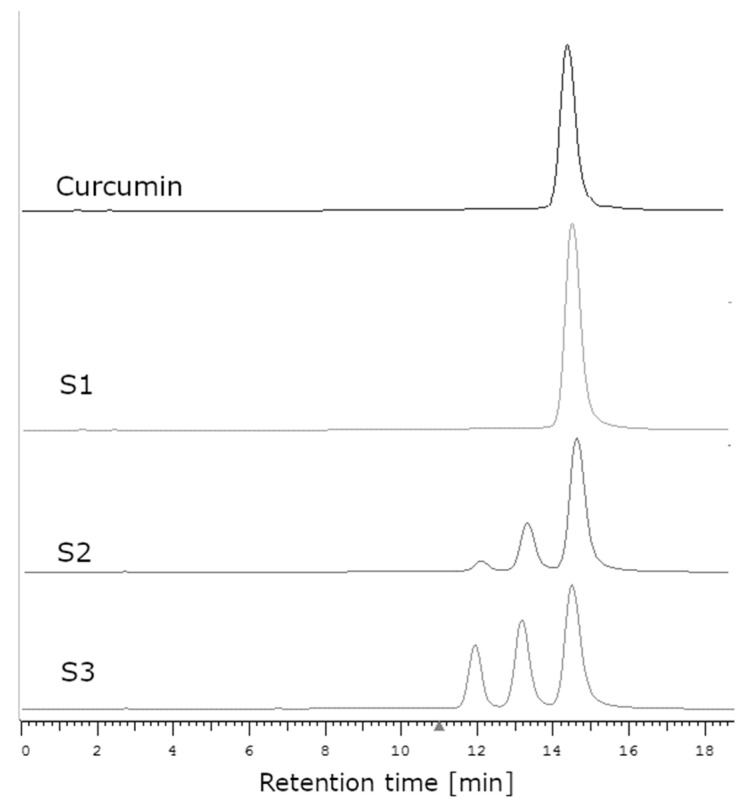
The chromatograms of curcumin standards (retention time, 14.7 min) and supplements S1–S3.

In order to verify the supposition that the examined S1 sample was faked by using pure synthetic curcumin instead of the genuine extract, the HPLC method was used. The registered chromatograms of samples S1, S2, and the curcumin standard are presented in Figure 8. 

In the case of the extract obtained from the plant, peaks derived from curcumin as well as from demethoxycurcumin (13.7 min) and bisdemethoxycurcumin (12 min) were observed on the chromatogram [44,45]. The purification of the extract in order to obtain pure curcumin without its derivatives is very laborious and time-consuming. The use of column chromatography makes it possible to obtain curcumin with a purity of 84–95% [46]. Crystallization allows one to obtain curcumin with a purity of about 99% [47]; using a combination of the crystallization and chromatographic methods, as much as 100% pure curcumin can be obtained. In the chromatogram of the curcumin standard, one intensive peak was visible (corresponding with curcumin) and another very small peak, which may have been the residue of curcumin derivatives (Figure 8). In the case of supplement S1, only the peak originating from curcumin was visible, which meant that the examined sample contained only curcumin with no derivatives. In the case of all other supplements (S2–S4), three peaks could be observed, corresponding with curcumin, demethoxycurcumin, and bisdemethoxycurcumin.

The raw plant material (curcumin) is the source of a complex of curcuminoids [46]. It is impossible to obtain an extract that would contain curcumin only without its derivatives. Such a degree of purity can only be reached using special purification methods (in particular, crystallization and column chromatography). In the case of the dietary supplements, this would considerably increase the costs of production; thus, it is rather unlikely that the manufacturer would carry out the purification of the extract to remove curcumin derivatives, especially as there was information on the package stating that the supplement contained the extract from turmeric rhizome standardized at 95% content of curcuminoids, including a minimum of 70% curcumin. Hence, it could be supposed that the manufacturer falsified the supplement by using synthetic curcumin. During synthesis, demethoxy and bisdemethoxy curcumin derivatives are not formed [48]. Synthetic curcumin is sold as a single molecule, with a lower cost compared with natural curcumin. The FDA has rejected the GRAS safety status for using synthetic curcumin in food [49].

So far, no scientific publications have been published that describe the application of ssNMR for a fast assessment of the quality of curcumin-containing dietary supplements. The ^1^H NMR technique in a solution can be successfully applied for a quantitative and qualitative investigation of curcumin-containing dietary supplements [50]. However, it requires an earlier dissolution of the sample in a solvent. The method proposed by us makes it possible to perform the analysis directly from the capsule content. Similarly, another study using the HPLC-PDA method to identify supplements containing synthetic curcumin required a sample extraction and special sample preparation [51].

## 3. Materials and Methods

Curcumin (CUR, C1386) was purchased from Sigma-Aldrich (Steinheim, Germany)). Four samples of curcumin-containing food supplements (S1–S4) were randomly selected from Polish pharmacies. A detailed description of the supplements provided by the manufacturers is included in Table 5.

Isopropyl alcohol was purchased from Avantor Performance Materials Poland S.A (Gliwice, Poland) and deuterated solvents (CDCl_3_ and DMSO-*d*_6_) from ARMAR Chemicals (Dottingen, Switzerland).

The ^1^H spectra for the CDCl_3_ and DMSO-*d*_6_ solutions were recorded at 300 MHz on a Varian VNMRS-300 spectrometer using a standard Varian pulse program. The ^13^C magic angle spinning (MAS) NMR spectra of solid samples were recorded on a Bruker DRX-400 Advance spectrometer using a Bruker PH MAS VTN 400WB BL4 probehead in the magnetic field of 9.4 T at 400.13 MHz (^1^H) and 100.62 MHz (^13^C). Samples were packed in a 4 mm ZrO_2_ rotor (Kel-F cap) and spun at 10 kHz. Standard ^13^C CPMAS spectra were obtained with a ^1^H 90° pulse length of 2.0 μs, with continuous wave (CW) proton decoupling. A contact time of 2 ms, a repetition time of 20 s, and a spectral width of 20 kHz were used for the accumulation of 800 scans for the standard ^13^C MAS experiments. ^13^C chemical shifts were calibrated indirectly through the glycine CO signal recorded at 176.50 ppm relative to TMS. The conventional ^1^H−^13^C CP pulse sequence with a reversal of the spin temperature in the rotating frame was applied with high-power proton decoupling during the signal acquisition. The dipolar dephased experiment was performed with a dipolar filter to suppress the ssNMR signals from the ^13^C nuclei, which were strongly coupled to protons (CH and CH_2_). Using a 60 µs delay before an acquisition caused the selective dephasing of the methine and methylene groups.

PXRD patterns were recorded using a Bruker D8 Advance diffractometer. Data were collected in the Bragg–Brentano geometry between 5° and 60° in steps of 0.25°. Data were collected under standard laboratory conditions of temperature and humidity.

The HPLC-DAD qualitative analysis of curcumin extracts was carried out using a Hitachi Chromaster HPLC system with DAD detector and a Purospher STAR RP-18 (5 μm, 4 × 250 mm) column at 30 °C. The analysis of curcumin in supplements was carried out under the following isocratic conditions: mobile phase, 50% acetonitrile with formic acid (0.1 vol%); flow rate, 1 mL/min; time, 0–25 min. The chromatograms were monitored at 430 nm. Figure 8 shows the chromatograms of curcumin standards (RT 14.7 min) and samples of supplements S1–S3 (Table 5).

The density functional theory (DFT) calculations of geometry optimization and NMR properties under periodic boundary conditions were carried out with the CASTEP program [52], implemented in Materials Studio 2017 software [53] using plane-wave pseudopotential formalism. 

On the fly-generated (OTFG) ultrasoft pseudopotentials were generated using the Koelling–Harmon scalar relativistic approach [54].

The experimental X-ray structure of curcumin Polymorph I (refcode BINMEQ05), Polymorph II (refcode BINMEQ06), and Polymorph III (BINMEQ07) from The Cambridge Structure Database (CSD) [37] were used as the initial for calculations. During geometry optimization, all atom positions and cell parameters were optimized. The convergence criteria were set at 5∙10^−6^ eV/atom for the energy, 1∙10^−2^ eV/Å for the interatomic forces, 2∙10^−2^ GPa for the stresses, and 5 10^−4^ Å for the maximum displacement. The fixed-basis set quality method for the cell optimization calculations and the 5 10^−7^ eV/atom tolerance for SCF were used.

The electronic parameter kinetic energy cutoff for the plane waves (Ecut) and number of Monkhorst–Pack k-points during sampling for a primitive cell Brillouin zone integration were set to 630.0 eV and 2 × 4 × 1 (for BINMEQ5), 1 × 3 × 2 (for BINMEQ6), and 2 × 3 × 1 (for BINMEQ7), respectively. 

The Perdew–Burke–Ernzerhof (PBE) [55] exchange-correlation functional, pure or with either a Tkatchenko–Scheffler (TS) [56] or Grimme [57] dispersion correction; the Perdew–Wang (PW91) exchange-correlation functional [58], pure or with the Ortmann–Bechstedt–Schmidt (OBS) [59] dispersion correction; the revised Perdew–Burke–Ernzerhof (RPBE) [60], Wu–Cohen (WC) [61], or solid design version of the PBE (PBESOL) [62] exchange-correlation functionals, defined within the generalized gradient approximation (GGA), were used as well as the local exchange-correlation functional of Perdew and Zunger [63] with the parameterization of the numerical results of Ceperley and Alder [64] (LDA CA-PZ), with or without the OBS method of dispersion correction.

The computation of shielding tensors was performed using the gauge-including projector augmented-wave (GIPAW) density functional theory method of Pickard et al. [65]. To compare the theoretical and experimental data, the calculated chemical shielding constants (σ_iso_) were converted to chemical shifts (δ_iso_) using the following equation: δ_iso_ = (σ_Gly_ + δ_Gly_) − σ_iso_, where σ_Gly_ and δ_Gly_ stand for the shielding constant and the experimental chemical shift, respectively, of the glycine carbonyl carbon atom (176.50 ppm).

## Figures and Tables

**Table 1:** The solubility of curcumin and its derivatives in ethanol, acetonitrile, and isopropanol.

	Ethanol	Acetonitrile	Isopropanol
	Solubility (g/L)		
Curcumin	3.9	7.2	0.91
Demethoxycurcumin	45	26	16.2
Bisdemethoxycurcumin	53	11	8.2

**Table 2:** Unit cell parameters obtained using different functionals.

Functional	a (Å)	b (Å)	c (Å)	α (°)	β (°)	γ (°)	Volume (Å^3^)
GGA PBESOL	12.375	7.567	20.771	90	90.510	90	1945.03
LDA CA-PZ	12.178	6.765	19.572	90	94.745	90	1607.00
LDA CA-PZ OBS	11.970	5.975	18.671	90	96.386	90	1327.06
GGA PBE	12.838	8.394	21.171	90	89.149	90	2281.16
GGA PBE TS	12.625	6.983	19.985	90	94.826	90	1755.64
GGA PBE Grimme	12.533	6.824	19.891	90	95.137	90	1694.48
GGA RPBE	13.479	9.988	22.124	90	86.774	90	2973.81
GGA PW91	13.001	8.631	21.321	90	88.326	90	2391.67
GGA PW91 OBS	12.273	6.523	19.207	90	96.289	90	1528.42
GGA WC	12.433	9.070	21.419	90	86.695	90	2411.54

**Table 3:** The experimental and theoretical chemical shift values (ppm) for three polymorphic forms of curcumin. Polymorph II * denotes the second molecule located in the independent part of the unit cell because for Polymorph II, Z` = 2.

C Atom Number	Experiment	Polymorph I	Polymorph II	Polymorph II *	Polymorph III
1	98.6	97.9	105.3	107.5	105.6
2	186.8	181.0	183.0	180.1	180.3
2’	182.4	174.4	171.8	172.9	171.9
3	122.8	122.5	120.1	119.6	121.0
3’	122.8	118.9	118.4	118.0	114.9
4	140.2	139.9	137.9	138.9	138.0
4’	140.2	138.7	135.2	136.5	138.1
5	128.8	126.3	125.1	122.7	121.6
5’	129.7	125.7	125.9	124.6	124.5
6	124.8	122.7	113.0	114.5	114.3
6’	124.8	120.1	112.7	114.5	113.1
7	114.1	109.2	110.0	112.0	114.4
7’	114.9	112.1	111.9	111.5	109.6
8	148.1	151.3	146.3	148.9	147.7
8’	148.1	149.5	145.1	146.8	147.4
9	148.1	145.7	144.2	145.8	145.7
9’	148.1	147.0	144.6	144.4	144.8
10	108.2	104.8	109.5	114.4	113.5
10’	109.8	112.1	111.9	111.5	109.6
11	54.9	49.8	51.0	52.2	51.4
11’	56.8	52.3	49.7	54.1	52.6
MAE		3.25	4.83	4.66	4.68

**Table 4:** The calculated differences between the experimental and theoretical values for each of the polymorphic forms. The blue color denotes the smallest values and red denotes the largest ones. Less intensive colors correspond with the smallest differences.

C Atom Number	δ_EXP_-δ_CALC_^I^	δ_EXP_-δ_CALC_^II^	δ_EXP_-δ_CALC_^II *^	δ_EXP_-δ_CALC_^III^
1	0.7	−6.7	−8.9	−7
2	5.8	3.8	6.7	6.5
2’	8	10.6	9.5	10.5
3	0.3	2.7	3.2	1.8
3’	3.9	4.4	4.8	7.9
4	0.3	2.3	1.3	2.2
4’	1.5	5	3.7	2.1
5	2.5	3.7	6.1	7.2
5’	4	3.8	5.1	5.2
6	2.1	11.8	10.3	10.5
6’	4.7	12.1	10.3	11.7
7	4.9	4.1	2.1	−0.3
7’	2.8	3	3.4	5.3
8	−3.2	1.8	−0.8	0.4
8’	−1.4	3	1.3	0.7
9	2.4	3.9	2.3	2.4
9’	1.1	3.5	3.7	3.3
10	3.4	−1.3	−6.2	−5.3
10’	−2.3	−2.1	−1.7	0.2
11	5.1	3.9	2.7	3.5
11’	4.5	7.1	2.7	4.2

II * denotes the second molecule located in the independent part of the unit cell because for Polymorph II, Z` = 2.

**Table 5:** Characteristics of dietary supplements used in the study.

No.	Form	Declared Content
S1	Capsule	250 mg turmeric rhizome extract (curcumin min. 70%); 2.5 mg black pepper extract (piperine 95%)
S2	Capsule	350 mg turmeric rhizome extract (266 mg curcumin); 5 mg black pepper extract (4.75 mg piperine)
S3	Capsule	332 mg curcumin (95%)
S4	Tablet	300 mg curcumin (95%); 5 mg piperine (95%)

## Data Availability

Data sharing not applicable.

## Supplementary Materials

The following supporting information can be downloaded at: https://www.mdpi.com/article/10.3390/molecules28083442/s1, Table S1: Crystallographic structures of curcumin deposited in The Cambridge Structural Database (CSD).

## Sample Availability

Samples of the examined food supplements are available from the authors.

## References

[1] Noorafshan A., Ashkani-Esfahani S. (2013). A review of therapeutic effects of curcumin. Curr. Pharm. Des..

[2] Pulido-Moran M., Moreno-Fernandez J., Ramirez-Tortosa C., Ramirez-Tortosa M. (2016). Curcumin and health. Molecules.

[3] Li N., Tian-Hao L., Jing-Ze Y., Chen-Xi L., Yang L., Yue-Ying W., Zhong-Shan Y., Jia-Li Y. (2019). Curcumin and curcumol inhibit NF-κB and TGF-β1/smads signaling pathways in CSE-treated RAW246. 7 cells. Evid. -Based Complement. Altern. Med..

[4] Vasanthkumar T., Hanumanthappa M., Lakshminarayana R. (2019). Curcumin and capsaicin modulates LPS induced expression of COX-2, IL-6 and TGF-β in human peripheral blood mononuclear cells. Cytotechnology.

[5] Banno A., Reddy A.T., Lakshmi S.P., Reddy R.C. (2018). PPARs: Key regulators of airway inflammation and potential therapeutic targets in asthma. Nucl. Recept. Res..

[6] Zhu T., Zhihong C., Guihua C., Daoxin W., Shuo T., Huojin D., Jing W., Shengjin L., Jian L., Jin T. (2019). Curcumin attenuates asthmatic airway inflammation and mucus hypersecretion involving a PPARγ-dependent NF-κB signaling pathway in vivo and in vitro. Mediat. Inflamm..

[7] Abdel-Daim M.M., Abdou R.H. (2015). Protective effects of diallyl sulfide and curcumin separately against thallium-induced toxicity in rats. Cell J..

[8] Al-Rubaei Z., Mohammad T.U., Ali L.K. (2014). Effects of local curcumin on oxidative stress and total antioxidant capacity in vivo study. Pak. J. Biol. Sci..

[9] Li Y., Jia L., Shanshan L., Yi L., Xiangxiang W., Baolin L., Qiang F., Shiping M. (2015). Curcumin attenuates glutamate neurotoxicity in the hippocampus by suppression of ER stress-associated TXNIP/NLRP3 inflammasome activation in a manner dependent on AMPK. Toxicol. Appl. Pharmacol..

[10] Giordano A., Tommonaro G. (2019). Curcumin and cancer. Nutrients.

[11] Stenzel A., Żuryń A., Grzanka A.A., Grzanka A. (2012). Cykliny jako markery chorób nowotworowych. Nowotwory. J. Oncol..

[12] Byun S.Y., Kim D.B., Kim E. (2015). Curcumin ameliorates the tumor-enhancing effects of a high-protein diet in an azoxymethane-induced mouse model of colon carcinogenesis. Nutr. Res..

[13] Mishra A., Kumar R., Tyagi A., Kohaar I., Hedau S., Bharti A.C., Sarker S., Dey D., Saluja D., Das B. (2015). Curcumin modulates cellular AP-1, NF-kB, and HPV16 E6 proteins in oral cancer. Ecancermedicalscience.

[14] Liu Y., Wang X., Zeng S., Zhang X., Zhao J., Zhang X., Chen X., Yang W., Yang Y., Dong Z. (2018). The natural polyphenol curcumin induces apoptosis by suppressing STAT3 signaling in esophageal squamous cell carcinoma. J. Exp. Clin. Cancer Res..

[15] Malik P., Hoidal J.R., Mukherjee T.K. (2021). Recent Advances in Curcumin Treated Non-Small Cell Lung Cancers: An Impetus of Pleiotropic Traits and Nanocarrier Aided Delivery. Curr. Med. Chem..

[16] Bayet-Robert M., Kwiatowski F., Leheurteur M., Gachon F., Planchat E., Abrial C., Mouret-Reynier M.A., Durando X., Barthomeuf C., Chollet P. (2010). Phase I dose escalation trial of docetaxel plus curcumin in patients with advanced and metastatic breast cancer. Cancer Biol. Ther..

[17] Kanai M., Yoshimura K., Asada M., Imaizumi A., Suzuki C., Matsumoto S., Nishimura T., Mori Y., Masui T., Kawaguchi Y. (2011). A phase I/II study of gemcitabine-based chemotherapy plus curcumin for patients with gemcitabine-resistant pancreatic cancer. Cancer Chemother. Pharmacol..

[18] Pastorelli D., Fabricio A.S.C., Giovanis P., D’Ippolito S., Fiduccia P., Soldà C., Buda A., Sperti C., Bardini R., Da Dalt G. (2018). Phytosome complex of curcumin as complementary therapy of advanced pancreatic cancer improves safety and efficacy of gemcitabine: Results of a prospective phase II trial. Pharmacol. Res..

[19] Kunnumakkara A.B., Harsha C., Banik K., Vikkurthi R., Sailo B.L., Bordoloi D., Gupta S.C., Aggarwalet B.B. (2019). Is curcumin bioavailability a problem in humans: Lessons from clinical trials. Expert Opin. Drug Metab. Toxicol..

[20] European Food Safety Authority (2014). Refined exposure assessment for curcumin (E 100). EFSA J..

[B21-molecules-28-03442] 21.FDA, FDA’s review of the physicochemical characteristics, safety, effectiveness, and historical use in compounding of curcumin presented to the Pharmacy Compounding Advisory Committee in October 2015 (“FDA’s curcumin review”).

[22] Górnicka J., Mika M., Wróbleska O., Siudem P., Paradowska K. (2023). Methods to Improve the Solubility of Curcumin from Turmeric. Life.

[23] Dwyer J.T., Coates P.M., Smith M.J. (2018). Dietary supplements: Regulatory challenges and research resources. Nutrients.

[24] Fibigr J., Šatínský D., Solich P. (2018). Current trends in the analysis and quality control of food supplements based on plant extracts. Anal. Chim. Acta.

[25] Dudek M.K., Kaźmierski S., Potrzebowski M.J. (2021). Fast and very fast MAS solid state NMR studies of pharmaceuticals. Annu. Rep. NMR Spectrosc..

[26] Li M., Xu W., Su Y. (2021). Solid-state NMR spectroscopy in pharmaceutical sciences. Trends Anal. Chem..

[27] Mathew R., Uchman K.A., Gkoura L., Pickard C.J., Baias M. (2020). Identifying aspirin polymorphs from combined DFT-based crystal structure prediction and solid-state NMR. Magn. Reson. Chem..

[28] Szeleszczuk Ł., Pisklak D.M., Gubica T., Matjakowska K., Kaźmierski S., Zielińska-Pisklak M. (2019). Application of combined solid-state NMR and DFT calculations for the study of piracetam polymorphism. Solid State Nucl. Magn. Reson..

[29] Czernek J., Brus J. (2020). Polymorphic forms of valinomycin investigated by NMR crystallography. Int. J. Mol. Sci..

[30] Dezena R.M. (2020). Ritonavir polymorphism: Analytical chemistry approach to problem solving in the pharmaceutical industry. Brazil. J. Anal. Chem..

[31] Bauer J., Spanton S., Henry R., Quick J., Dziki W., Porter W., Morris J. (2001). Ritonavir: An extraordinary example of conformational polymorphism. Pharm. Res..

[32] Dąbrowska-Balcerzak K., Nartowska J., Wawer I., Siudem P., Paradowska K. (2021). Spirostanol sapogenins and saponins from Convallaria majalis L. structural characterization by 2D NMR, theoretical GIAO DFT calculations and molecular modeling. Molecules.

[33] Siudem P., Paradowska K., Bukowicki J. (2017). Conformational analysis of capsaicin using 13C, 15N MAS NMR, GIAO DFT and GA calculations. J. Mol. Struct..

[34] Siudem P., Bukowicki J., Wawer I., Paradowska K. (2020). Structural studies of two capsaicinoids: Dihydrocapsaicin and nonivamide. 13 C and 15 N MAS NMR supported by genetic algorithm and GIAO DFT calculations. RSC Adv..

[35] Presti D., Pedone A., Menziani M.C. (2014). Unraveling the polymorphism of [(p-cymene) Ru (κN-INA) Cl2] through dispersion-corrected DFT and NMR GIPAW calculations. Inorg. Chem..

[36] Marín-Luna M., Alkorta I., Elguero J. (2018). A theoretical NMR study of selected benzazoles: Comparison of GIPAW and GIAO-PCM (DMSO) calculations. Magn. Reson. Chem..

[37] The Cambridge Structural Database. https://www.ccdc.cam.ac.uk/.

[38] Cui Z., Yao L., Ye J., Wang Z., Hu Y. (2021). Solubility measurement and thermodynamic modelling of curcumin in twelve pure solvents and three binary solvents at different temperature (T= 278.15–323.15 K). J. Mol. Liq..

[39] Sanphui P., Goud N.R., Khandavilli U.R., Bhanoth S., Nangia A. (2011). New polymorphs of curcumin. Chem. Comm..

[40] Matlinska M.A., Wasylishen R.E., Bernard G.M., Terskikh V.V., Brinkmann A., Michaelis V.K. (2018). Capturing elusive polymorphs of curcumin: A structural characterization and computational study. Cryst. Growth Design.

[41] Kolev T.M., Velcheva E.A., Stamboliyska B.A., Spiteller M. (2005). DFT and experimental studies of the structure and vibrational spectra of curcumin. Int. J. Quantum Chem..

[42] Byard S.J., Jackson S.L., Smail A., Bauer M., Apperley D.C. (2005). Studies on the crystallinity of a pharmaceutical development drug substance. J. Pharm. Sci..

[43] Pisklak D.M., Zielińska-Pisklak M.A., Szeleszczuk Ł., Wawer I. (2016). ^13^C solid-state NMR analysis of the most common pharmaceutical excipients used in solid drug formulations, Part I: Chemical shifts assignment. J. Pharm. Biomed. Anal..

[44] Peram M.R., Jalalpure S.S., Joshi S.A., Palkar M.B., Diwan P.V. (2017). Single robust RP-HPLC analytical method for quantification of curcuminoids in commercial turmeric products, Ayurvedic medicines, and nanovesicular systems. J. Liq. Chromatogr. Relat. Technol..

[45] Heffernan C., Ukrainczyk M., Gamidi R.K., Hodnett B.K., Rasmuson Å.C. (2017). Extraction and purification of curcuminoids from crude curcumin by a combination of crystallization and chromatography. Org. Process. Res. Dev..

[46] Jiang T., Ghosh R., Charcosset C. (2021). Extraction, purification and applications of curcumin from plant materials-A comprehensive review. Trends Food Sci. Technol..

[47] Ukrainczyk M., Hodnett B.K., Rasmuson A.C. (2016). Process parameters in the purification of curcumin by cooling crystallization. Org. Process. Res. Dev..

[48] Bruzell E.M., Morisbak E., Tønnesen H.H. (2005). Studies on curcumin and curcuminoids. XXIX. Photoinduced cytotoxicity of curcumin in selected aqueous preparations. Photochem. Photobiol. Sci..

[49] The Food and Drug Administration. https://www.fda.gov/media/130730/download/.

[50] Sorng S., Balayssac S., Danoun S., Assemat G., Mirre A., Cristofoli V., Le Lamer A.C., Jullian V., Gilard V., Fabre N. (2022). Quality assessment of Curcuma dietary supplements: Complementary data from LC-MS and ^1^H NMR. J. Pharm. Biomed. Anal..

[51] Girme A., Saste G., Balasubramaniam A.K., Pawar S., Ghule C., Hingorani L. (2020). Assessment of Curcuma longa extract for adulteration with synthetic curcumin by analytical investigations. J. Pharm. Biomed. Anal..

[52] Clark S.J., Segall M.D., Pickard C.J., Hasnip P.J., Probert M.J., Refson K., Payne M.C.Z. (2005). First principles methods using CASTEP. Für Krist. -Cryst. Mater..

[53] BIOVIA Materials Studio. https://www.3ds.com/products-services/biovia/products/molecular-modeling-simulation/biovia-materials-studio/.

[54] Koelling D.D., Harmon B.N. (1977). Technique for relativistic spin-polarized calculations. J. Phys. C Solid State Phys..

[55] Perdew J.P., Burke K., Ernzerhof M. (1996). Generalized Gradient Approximation Made Simple. M. Phys. Rev. Lett..

[56] Tkatchenko A., Scheffler M. (2009). Accurate Molecular Van Der Waals Interactions from Ground-State Electron Density and Free-Atom Reference Data. M. Phys. Rev. Lett..

[57] Grimme S. (2006). Semiempirical GGA-type density functional constructed with a long-range dispersion correction. J. Comput. Chem..

[58] Perdew J.P., Chevary J.A., Vosko S.H., Jackson K.A., Pederson M.R., Singh D.J., Fiolhais C. (1992). Atoms, molecules, solids, and surfaces: Applications of the generalized gradient approximation for exchange and correlation. Phys. Rev. B.

[59] Ortmann F., Bechstedt F., Schmidt W.G. (2006). Semiempirical van der Waals correction to the density functional description of solids and molecular structures. Phys. Rev. B..

[60] Hammer B., Hansen L.B., Norskov J.K. (1999). Improved adsorption energetics within density-functional theory using revised Perdew-Burke-Ernzerhof functionals. Phys. Rev. B..

[61] Wu Z., Cohen R.E. (2006). More accurate generalized gradient approximation for solids. Phys. Rev. B..

[62] Perdew J.P., Ruzsinszky A., Csonka G.I., Vydrov O.A., Scuseria G.E., Constantin L.A., Zhou X., Burke K. (2008). Restoring the Density-Gradient Expansion for Exchange in Solids and Surfaces. Phys. Rev. Lett..

[63] Perdew J.P., Zunger A. (1981). Self-interaction correction to density-functional approximations for many-electron systems. Phys. Rev. B..

[64] Ceperley D.M., Alder B.J. (1980). Ground State of the Electron Gas by a Stochastic Method. Phys. Rev. Lett..

[65] Pickard C.J., Mauri F. (2001). All-electron magnetic response with pseudopotentials: NMR chemical shifts. Phys. Rev. B..

