# Supplementary material for: 13C CPMAS NMR as an Alternative Method to Verify the Quality of Dietary Supplements Containing Curcumin
Source: Molecules. 2023 Apr 13;28(8):3442. doi: 10.3390/molecules28083442 (PMC10143288; doi:10.3390/molecules28083442)
Supplement: Supplementary file 1 [file molecules-28-03442-s001.zip › molecules-2304252-supplementary.pdf]

Table S1. Crystallographic structures of curcumin deposited in The Cambridge Structural Database (CSD).

Ref. code	Group	Form	a [Å]	b [Å]	c [Å]	α [⁰]	β [⁰]	γ [⁰]	Volume [Å³]	Z	Z′	Temp [⁰C]
BINMEQ	P2/n	I	20.028	7.073	12.609	90	94.94	90	1779.53	4	1	121
BINMEQ1	P2/n	I	12.688	7.206	19.897	90	95..28	90	1811.48	4	1	283-303
BINMEQ2	P2/n	I	12.707	7.219	19.880	90	95.35	90	1815.59	4	1	283-303
BINMEQ3	P2/n	I	12.695	7.207	19.960	90	95.10	90	1819.10	4	1	283-303
BINMEQ4	P2/n	I	12.584	7.039	19.951	90	95.04	90	1760.49	4	1	90
**BINMEQ5**	**P2/n**	**I**	**12.568**	**7.042**	**19.958**	**90**	**94.99**	**90**	**1759.76**	**4**	1	**100**
**BINMEQ6**	**Pca2₁**	**II**	**35.417**	**7.779**	**12.648**	**90**	**90**	**90**	**3484.78**	**8**	2	**100**
**BINMEQ7**	**Pbca**	**III**	**12.536**	**7.992**	**34.462**	**90**	**90**	**90**	**3452.50**	**8**	1	**100**
BINMEQ8	Pca2₁	II	35.537	7.780	12.680	90	90	90	3505.56	8	2	283-303
BINMEQ9	P2/n	I	12.697	7.198	19.953	90	95.12	90	1816.36	4	1	283-303
BINMEQ10	P2/n	I	12.685	7.184	19.895	90	95.22	90	1805.71	4	1	283-303
BINMEQ11	P2/n	I	12.626	7.110	19.956	90	95.08	90	1784.50	4	1	173
BINMEQ12	Pca2₁	II	35.446	7.850	19.693	90	90	90	3531.70	8	2	173
BINMEQ13	P2/n	I	12.683	7.177	19.916	90	95.05	90	1805.63	4	1	283-303
BINMEQ14	P2/n	I	12.695	7.209	19.936	90	95.28	90	1816.96	4	1	283-303
